# OsJAZ1 Attenuates Drought Resistance by Regulating JA and ABA Signaling in Rice

**DOI:** 10.3389/fpls.2017.02108

**Published:** 2017-12-11

**Authors:** Jie Fu, Hua Wu, Siqi Ma, Denghao Xiang, Ruyi Liu, Lizhong Xiong

**Affiliations:** National Key Laboratory of Crop Genetic Improvement and National Center of Plant Gene Research (Wuhan), Huazhong Agricultural University, Wuhan, China

**Keywords:** jasmonates, abscisic acid, JAZ protein, crosstalk, drought resistance

## Abstract

Jasmonates (JAs) and abscisic acid (ABA) are phytohormones known play important roles in plant response and adaptation to various abiotic stresses including salinity, drought, wounding, and cold. JAZ (JASMONATE ZIM-domain) proteins have been reported to play negative roles in JA signaling. However, direct evidence is still lacking that JAZ proteins regulate drought resistance. In this study, OsJAZ1 was investigated for its role in drought resistance in rice. Expression of *OsJAZ1* was strongly responsive to JA treatment, and it was slightly responsive to ABA, salicylic acid, and abiotic stresses including drought, salinity, and cold. The *OsJAZ1*-overexpression rice plants were more sensitive to drought stress treatment than the wild-type (WT) rice Zhonghua 11 (ZH11) at both the seedling and reproductive stages, while the *jaz1* T-DNA insertion mutant plants showed increased drought tolerance compared to the WT plants. The *OsJAZ1*-overexpression plants were hyposensitive to MeJA and ABA, whereas the *jaz1* mutant plants were hypersensitive to MeJA and ABA. In addition, there were significant differences in shoot and root length between the OsJAZ1 transgenic and WT plants under the MeJA and ABA treatments. A subcellular localization assay indicated that OsJAZ1 was localized in both the nucleus and cytoplasm. Transcriptome profiling analysis by RNA-seq revealed that the expression levels of many genes in the ABA and JA signaling pathways exhibited significant differences between the *OsJAZ1*-overexpression plants and WT ZH11 under drought stress treatment. Quantitative real-time PCR confirmed the expression profiles of some of the differentially expressed genes, including *OsNCED4, OsLEA3, RAB21, OsbHLH006, OsbHLH148, OsDREB1A, OsDREB1B, SNAC1*, and *OsCCD1*. These results together suggest that OsJAZ1 plays a role in regulating the drought resistance of rice partially via the ABA and JA pathways.

## Introduction

Due to their sessile nature, plants have to face variable environmental stresses including drought, high salinity, cold, and heat during their growth and development. Plants respond and adapt to the adverse cues throughout their life cycle by coordinating an array of biochemical and physiological changes. Phytohormones, such as abscisic acid (ABA) and jasmonates (fatty acid-derived oxylipins, JAs), play important roles in promoting plant defense against abiotic stresses (Verma et al., 2016).

To date, key components of JA perception and signaling have been extensively studied in numerous plant species, particularly in *Arabidopsis*, tomato (*Solanum lycopersicum*), and rice (*Oryza sativa*) (Cheong and Choi, 2003; Goossens et al., 2016; Wasternack and Song, 2017). According to the updated model of JA perception and the regulation of JA-responsive genes (Xu, 2002; Chini et al., 2007; Sheard et al., 2010; Zhu et al., 2011), the COI1-JAZ complex acts as JA co-receptor and transcriptional repressor in JA signaling. Under normal or JA-absent conditions, JAZ proteins bind to transcription factors (TFs) and inhibit their activities by recruiting the TOPLESS (TPL) co-repressor or by directly recruiting histone-modifying proteins such as HDA6. These TFs can bind to the G-box of JA- and JA-Ile-responsive genes. Under any stress conditions or developmental processes in which the levels of endogenous JA-Ile (bioactive jasmonyl-isoleucine) are increased, the binding of JA-Ile to the COI1-JAZ co-receptor will lead to the degradation of JAZs via the 26S proteasome, and the JA signaling responses and downstream genes are activated.

JASMONATE ZIM-domain (JAZ) proteins, belonging to the group II of the TIFY family, have been identified as important regulators of JA signaling in numerous plants, including *Arabidopsis thaliana, Solanum lycopersicum*, and rice, etc. There are 20 and 18 members of the TIFY family in rice and *Arabidopsis*, respectively (Vanholme et al., 2007; Ye et al., 2009). In previous studies, overexpression of *JAZ1* lacking the Jas domain in *Arabidopsis* showed an obvious JA-hyposensitive phenotype, and a similar phenotype was observed in a *JAI3/JAZ* mutant (Chini et al., 2007; Thines et al., 2007). Furthermore, JAZ proteins have been well characterized for their interaction with bHLH TFs to repress the transcriptional activities of bHLH, which are the core transcriptional activators of JA signaling mediated gene expression. For example, MYC2, MYC3, and MYC4, which are core bHLH factors in the regulation of JA signaling in rice and *Arabidopsis*, have been reported to interact with almost all the JAZ proteins (Cheng et al., 2011; Fernandez-Calvo et al., 2011; Sasaki-Sekimoto et al., 2014). JAZ proteins are also found to interact with the F-box protein COI1, leading to the degradation of the JAZ proteins (Song et al., 2011). The *coi1* mutants in *Arabidopsis* were defective in the JA response. In addition, expression of the JA-induced genes *AtVSP, Thi2.0*, and *PDF1.2*, which encode defense-related proteins, were suppressed in the *coi1* mutant (Penninckx et al., 1998; Xu et al., 2001), indicating that *COI1* is a core player in the JA signaling pathway. JAZ proteins have been reported to be involved in the regulation of trichome formation, anthocyanin synthesis, and male fertility by interacting with various TFs and affecting their transcriptional function (Kirik et al., 2005; Gonzalez et al., 2008; Qi et al., 2011; Song et al., 2011). JAZ proteins directly interacted with a wide array of TFs (such as GL3, EGL3, TT8, and the MYB factor MYB75), repressed their transcriptional function, and then suppressed JA-induced anthocyanin biosynthesis and trichome initiation (Kirik et al., 2005; Gonzalez et al., 2008; Qi et al., 2011). In addition, JAZ proteins (JAZ1, JAZ8, and JAZ11) in *Arabidopsis* were reported to interact with two R2R3-MYB TFs MYB21 and MYB24, which inhibited the expression of downstream genes essential for JA-mediated stamen development (Song et al., 2011). JAZ proteins also have been reported to be involved in the regulation of abiotic stresses including cold, salinity, drought, wounding, and ozone (Savchenko et al., 2014; Wu et al., 2015). Hu et al. (2013) suggested that JAZ proteins (JAZ1 and JAZ4) physically interact with ICE1, resulting in a blockage in the ICE-CBF pathway in *Arabidopsis*. JAZ9, a transcriptional regulator in JA signaling, is known to modulate salt tolerance in rice (Wu et al., 2015).

Abscisic acid also plays an important role in regulating various stress responses and the expression of stress responsive genes (Stone et al., 2006; Nilson and Assmann, 2007; Sirichandra et al., 2009). A number of studies have shown that JA signaling can interact with ABA signaling (Lackman et al., 2011; Chen et al., 2012; Aleman et al., 2016). *MED25* plays a positive role in JA signaling via *OsMYC2*, while it negatively regulates the protein abundance of ABI5, which plays a core role in ABA signaling (Chen et al., 2012). In addition, *HDA6*, an RPD3-type histone deacetylase, was found to be involved in the JA and ABA response (Wu et al., 2008; Chen and Wu, 2010). PYL4, an ABA receptor, is involved in the crosstalk between JA and the ABA pathway to regulate plant metabolism and growth (Lackman et al., 2011). The ABA-dependent signaling pathway is partially controlled by MYC/MYB TFs, which are target proteins of JAZ proteins. It has been demonstrated that ATMYC2 (JIN1) in *Arabidopsis*, an important player in JA signaling, can function as a positive activator in the expression of ABA and the drought inducible gene *RD22* (Abe et al., 2003). Recently, several publications have demonstrated that JAs are involved in the regulation of numerous stress-responsive genes which are also regulated by ABA. The expression levels of *SNAC1* and *OsbHLH148*, involved in the regulation of drought-responsive genes, were both increased by MeJA and ABA treatments (Takasaki et al., 2010; Seo et al., 2011). The crosstalk between JA and ABA signaling was also observed in guard cells. CPK6, an *Arabidopsis* calcium dependent protein kinase, was involved in both ABA and JA signaling in guard cells (Munemasa et al., 2011). Moreover, de Ollas et al. (2015) suggested that the ABA concentration was increased fourfold to sevenfold in an exogenous JA treatment, while a JA deficiency mutant *jarl* showed reduced ABA accumulation.

Although previous studies clearly suggest that JA is involved in drought responses, direct evidence for JAZ proteins in regulating drought resistance is lacking. In this study, we aimed to investigate whether and how a stress-responsive JAZ gene, *OsJAZ1*, regulates drought resistance in rice. The *OsJAZ1-*overexpression (*OsJAZ1-*OE) plants showed increased sensitivity to drought stress, while the *jaz1* mutant plants were more hyposensitive to drought stress compared to the wild-type (WT) plants. Moreover, the *jaz1* mutant plants were more hypersensitive to MeJA and ABA, indicating that JAZ1 may play a negative role in modulating JA and ABA signaling. Consistent with this, many genes in the JA and ABA signaling pathways were affected by *OsJAZ1* overexpression under drought stress conditions. Our results suggest that OsJAZ1 plays a negative role in drought resistance, partially through regulation of the JA and ABA pathways.

## Materials and Methods

### Plant Materials and Growth Conditions

The *OsJAZ1* mutant line 4A-00845 in the background of the rice variety Dongjin (*Oryza sativa* L. ssp. *japonica*) was obtained from the POSTECH RISD^1^. Homozygous mutant lines (mut-1 and mut-2) were segregated from the heterozygous mutant 4A-00845. Genotyping was performed using the *OsJAZ1* genomic primers and the T-DNA left-border primer (Supplementary Table 1). The full coding sequence of *OsJAZ1* (LOC_Os04g55920.1) was amplified from the total cDNA of rice leaves. The amplified fragment of *OsJAZ1* was cloned into pCAMBIA1301H, which was driven by the *OsLEA3* promoter (Xiao et al., 2007). The plasmid was then introduced into the *japonica* rice cultivar Zhonghua11 (ZH11) via *Agrobacterium*-mediated transformation (Hiei and Komari, 2008). The rice plants used for drought resistance testing were grown in pots or paddy field under the natural conditions at Wuhan (114.36°E, 30.48°N), China. The seedlings for phytohormone treatments were grown in a phytotron (PPFD 75 μmol/m^2^.s) with a 14 h light/10 h dark cycle for 7 days with a temperature at 28°C and 25°C, for the light and dark conditions, respectively.

### Drought Resistance Testing

To investigate the drought stress resistance of *OsJAZ1*-OE plants and *jaz1* mutant plants at the seedling stage, WT and transgenic seeds were germinated on 1/2 strength MS medium for 3 days in the dark and 4 days in a greenhouse. WT and transgenic lines were transplanted into barrels (30 cm in diameter and 25 cm in height) filled with a mixture of sand and soil (1:1). Each barrel was planted with about 10 transgenic seedlings and 10 WT seedlings in a half-and-half manner. Seedlings at the four-leaf stage were subjected to drought stress treatment for 4 days with the water content of the soil maintained at 5–10%. After recovery by watering for 7 days, the plants were photographed and the survival rates were recorded. Each of the OE or mutant lines were tested with three biological repeats for drought resistance. Drought stress treatment was also performed in the barrels at the reproductive stage. In this testing, each barrel was planted with a single plant. A rice automatic phenotyping (RAP) platform (Yang et al., 2014) was used to extract phenotypic traits of the plants in the barrels. At the booting stage, the WT and transgenic plants were subjected to drought stress treatment for 7 days with the soil moisture content maintained at 15%. All the materials in this had three repeats under both normal growth and drought stress conditions. To reflect the morphological changes of plants in response to drought stress treatment, we adopted an image index called PAR, which was defined as the plant perimeter divided by the projected plant area. Since rice leaves exhibit rolling under moderate and severe drought stress conditions, the projected area will decrease, but the plant perimeter will not change too much. Therefore, the change in PAR will largely reflect the degree of leaf rolling and plant architecture. Drought stress treatment at the reproductive stage was performed in a refined paddy field facilitated with a movable rain-off shelter. Each material was planted in a plot of 10 plants with three repeats. The transgenic plants and WT plants at the booting stage were subjected to drought stress treatment for about 3 weeks with the soil moisture content maintained at 15% for the final 5 days. Then the field was fully irrigated for recovery. The relative water content (RWC) in plant leaves were determined as follows: RWC % = 100% × (fresh weight – dry weight)/(turgid weight – dry weight). The dry weight was determined by placing the samples at 80°C for 24 h. Turgid weight was obtained after placing the samples in ddH_2_O at room temperature for 3 h.

### Phytohormone Treatment

**FIGURE 1:**
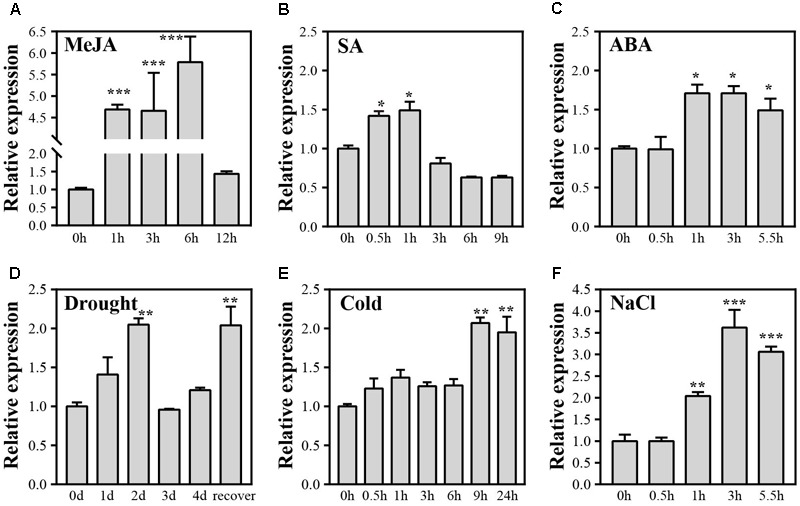
Expression levels of *OsJAZ1* under various abiotic stresses and phytohormone treatments. **(A–F)** Seedlings at the four-leaf stage were subjected to treatment with drought stress, NaCl (200 mmol/L), cold (4°C), ABA (100 μmol/L), MeJA (100 μmol/L), and SA (100 μmol/L), respectively. Error bars represent the SE of three replicates. Significance was determined by Student’s *t*-test (^∗∗∗^*P* < 0.001, ^∗∗^*P* < 0.01, ^∗^*P* < 0.05).

To test the phytohormones (ABA and JA) sensitivity of the *OsJAZ1*-OE transgenic plants and T-DNA mutant plants at the seedling stage, WT plants and transgenic lines were germinated on 1/2 strength MS medium for 3 days in the dark. After germination, seedlings with similar shoot length and root length were transplanted to transparent plastic plates (13 cm × 13 cm, seven plants half by half, at least three repeats) with 1/2 strength MS medium containing phytohormones (3 μM for ABA, 10 μM for MeJA) or water as a control. Finally, the phenotypes were recorded and shoot length and root length of these seedling were measured. To check the expression level of *OsJAZ1* under phytohormone treatments, the seedlings at four-leaf stage were sprayed with ABA (100 μmol/L), MeJA (100 μmol/L), and SA (100 μmol/L), respectively, each with three replicates. Then seedling leaves were sampled in a designed time course indicated in **Figure 1**. The concentration for testing MeJA and ABA sensitivity of OsJAZ1-OE and T-DNA mutant plants were selected by referring to Cai et al. (2014) and Tang et al. (2016).

### Subcellular Localization

For investigating the subcellular localization of the OsJAZ1 protein, the coding region of *OsJAZ1* was cloned into the PM999-35 vector with C-terminal YFP tag by a one-step *in vitro* recombination method (Gibson et al., 2009). The resulting construct (35S: JAZ1-YFP) and the nuclear marker 35S: CFP-GHD7 (Xue et al., 2008) were used for transient transformation of protoplasts. The protocol for isolation and transformation of rice protoplasts was described previously (Xie and Yang, 2013). The fluorescence signal was observed with a confocal laser-scanning microscope (FV1200) at 16–18 h after transformation. To confirm the nuclear signal of JAZ1, the protoplasts were treated with 100 μM MeJA for 3 h in the dark before collection for microscope observation.

### RNA Extraction and Quantification of Gene Expression

All the harvested leaf samples were frozen in liquid nitrogen, and RNA was extracted using TRIzol^®^ reagent (Ambion^TM^, Lot No.15596018) according to the manufacturer’s instructions. RNA was reverse transcribed using the EasyScript One-Step gDNA Removal and cDNA Synthesis Kit (TRANS) according to the manufacturer’s protocol. The quality of cDNA product was checked by amplifying *OsActin* (LOC_Os03g50885), which was used as an internal control and then the cDNA sample was 10-fold diluted with ddH_2_O for qRT-PCR. Real-time PCR was performed using a Quant Studio 6 Flex Real-time system with a SYBR Green Master Mix kit (Applied Biosystems^TM^). Three repeated reactions were routinely performed for each sample. The PCR reactions were performed as follows: 50°C for 2 min, 95°C for 2 min, followed by 40 cycles of 95°C for 1 s, 60°C for 30 s. All primers used in this study for qPCR are shown in Supplementary Table 1. The quantification of the relative expression level followed the reported 2^-ΔΔC_T_^ method (Livak and Schmittgen, 2001).

### RNA-Seq and Transcriptome Profiling Analysis

Total RNA was extracted from WT Zhonghua 11 (ZH11) and the *OsJAZ1-*OE plants at the four-leaf stage under normal and drought stress conditions with three biological replicates. The RNA samples were sequenced by Novogene (Tianjin, China) with Hiseq-PE150 and the raw data was analyzed using the customized RNA-seq data processing platform at the BMKCloud cloud server^2^. According to the results of Pearson’s correlation coefficient (Schulze et al., 2012), the third biological replicate of WT ZH11 and *OsJAZ1*-OE under drought stress was excluded from further analysis because of a low correlation coefficient. The expression levels of the genes were quantified by FPKM (fragments per kilobase of transcript per million fragments mapped) (Florea et al., 2013). The fold change of the expression level of the stressed sample over the corresponding non-stressed sample was calculated, and the genes with an absolute value of |log_2_(fold change)|≥ 1 and FDR < 0.05 were selected as differentially expressed genes (DEGs) (Anders and Huber, 2010). The Gene Ontology Consortium and Kyoto Encyclopedia of Genes and Genomes were referenced by GO enrichment analysis and the functional associated pathways, respectively, for the DEGs.

The RNA-Seq data were deposited in the Gene Expression Omnibus under accession number GSE107425.

### Statistical Analysis

The Student’s *t*-test was used for statistical analysis. Statistical significance was determined at *P* ^∗^< 0.05, *P* ^∗∗^< 0.01, and *P* ^∗∗∗^< 0.001.

## Results

### Expression Analysis of *OsJAZ1*

To investigate if *OsJAZ1* is involved in stress response, we applied quantitative real-time PCR (qPCR) to check the expression profiles of *OsJAZ1* under various phytohormone treatments and abiotic stresses at the four-leaf stage of rice. The results showed that *OsJAZ1* was strongly induced by MeJA (**Figure 1A**), suggesting that JAZ1 may be involved in JA signaling. *OsJAZ1* was slightly up-regulated by exogenous SA and ABA (**Figures 1B,C**). *OsJAZ1* was also induced by multiple abiotic stresses including drought, cold, and salinity (**Figures 1D–F**). Considering the biochemical function of JAZ proteins, these results suggest that OsJAZ1 may participate in the regulation of the responses to abiotic stresses and multiple phytohormones.

### *OsJAZ1* Negatively Regulates Drought Resistance at the Seedling Stage

**FIGURE 2:**
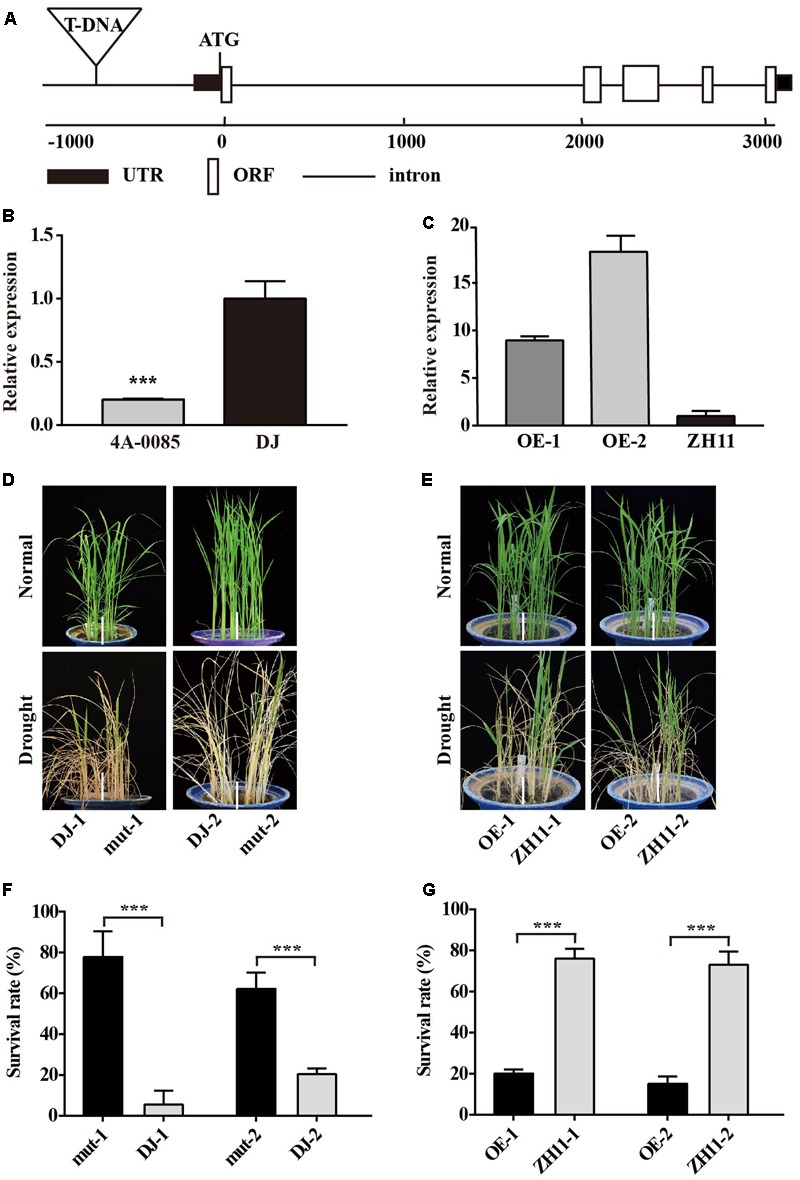
Phenotypes of the *OsJAZ1*-OE plants and the *jaz1* T-DNA insertion mutant plants under drought stress treatment at the seedling stage. **(A)** Schematic diagram of the *OsJAZ1* gene and 4A-00845 T-DNA insertion mutant. UTR, ORF, and introns are indicated in the black box, white box, and line, respectively. **(B)** Expression levels of *OsJAZ1* in the 4A-00845 homozygous line and Dongjin. **(C)** The expression levels of *OsJAZ1* in the OE lines and WT ZH11. **(D,E)** Drought tolerance testing of *jaz1* T-DNA mutant plants and over-expression plants. **(D)** The *jaz1* mutant lines showed increased drought resistance. **(E)** The *OsJAZ1*-OE plants were more sensitive to drought stress treatment compared to WT ZH11. **(F,G)** The survival rates of the transgenic and WT plants after recovery from drought stress treatment for 7 days. Mut-1 and mut-2 were heterozygous mutants segregated from 4A-00845. Error bars represent the SE of three replicates (^∗∗∗^*P* < 0.005, Student’s *t*-test).

To answer whether OsJAZ1 is involved in drought resistance regulation, we requested and obtained a heterozygous mutant 4A-00845 (Dongjin rice background) from the POSTECH RISD^3^. As shown in **Figure 2A**, the T-DNA was inserted in the promoter of *OsJAZ1*, and homozygous mutant plants (mut-1 and mut-2) were identified by PCR analysis using the primers Z1-mutF, Z1-mutR, and PGAR (Supplementary Table 1). The expression level of *OsJAZ1* in the mutant was dramatically repressed (**Figure 2B**). The primers JAZ1rtF and JAZ1rtR (Supplementary Table 1) were used for *OsJAZ1* expression analysis. For testing drought resistance at the seedling stage, the jaz1 mutant plants and the WT Dongjin plants of 3–4 cm length were transplanted to a blue barrel which was filled with a mixture of sand and soil (1:1). The mutant lines and WT seedlings were subjected to drought stress at the four-leaf stage. The water content of the soil was maintained at 5–10% for 3–4 days, and then watered for recovery. All the *jaz1* mutant lines (mut-1 and mut-2) showed increased tolerance to drought stress treatment (**Figure 2D**). After recovery for 7 days, about 80% of the *jaz1* mutant plants survived, while only 10–20% of the WT plants survived (**Figure 2F**). This result suggested that JAZ1 may have a negative role in drought resistance.

To further confirm the negative role of JAZ1 in drought resistance, we constructed an overexpression vector of *OsJAZ1* which was driven by the drought-inducible *OsLEA3-1* promoter (Xiao et al., 2007). We selected two independent *OsJAZ1*-OE transgenic lines (ZH11 background) with the expression levels of *OsJAZ1* in the transgenic plants confirmed by quantitative real-time PCR (**Figure 2C**). The *OsJAZ1*-OE plants and WT ZH11 were treated with drought stress at the four-leaf stage. During the drought stress treatment, the *OsJAZ1*-OE lines were more sensitive to the stress compared to the WT ZH11 (**Figure 2E**). After recovery, the survival rate of the *OsJAZ1*-OE lines (20%) was significantly lower than that of ZH11 (80%) (**Figure 2G**). These results together further supported that JAZ1 may act as a negative regulator in drought resistance.

### *OsJAZ1* Negatively Affects Drought Resistance at the Reproductive Stage

**FIGURE 3:**
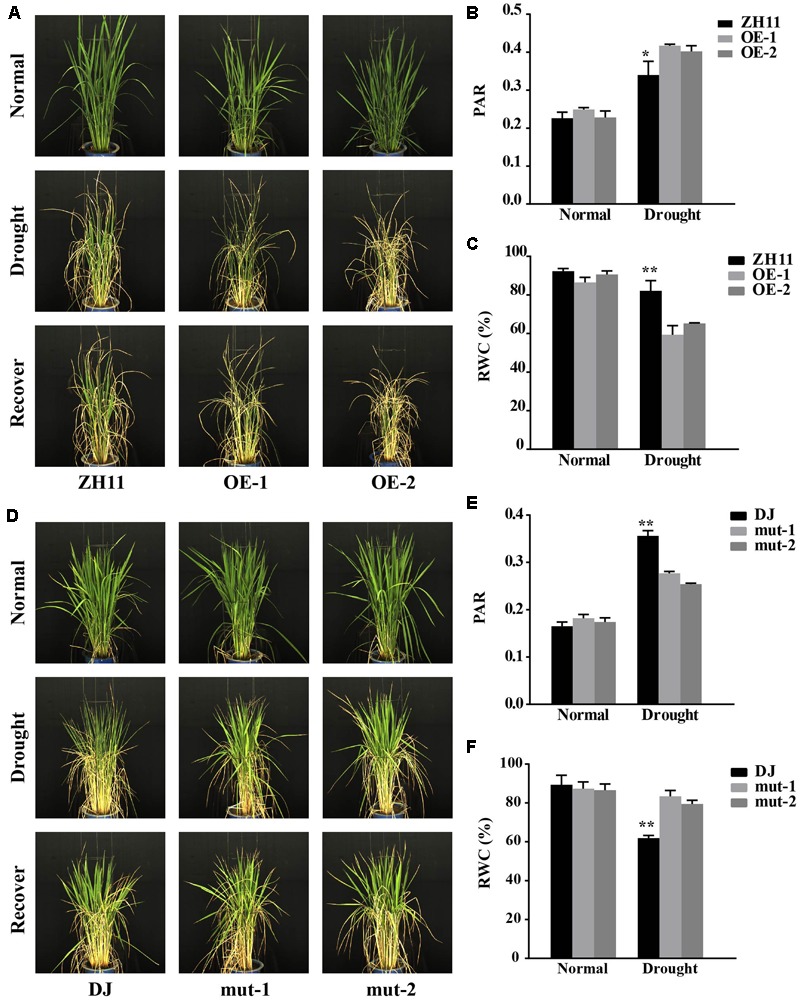
Drought resistance testing of the *OsJAZ1*-OE and mutant lines at the reproductive stage. **(A)** The *OsJAZ1*-OE lines and the WT plants grown in barrels at the booting stage were subjected to drought stress treatment for 7 days with the soil moisture content maintained at 15%. The degree of leaf rolling is indicated by the PAR **(B)** and the relative water content (RWC) **(C)** of the *OsJAZ1*-OE lines under normal and drought stress conditions. PAR = plant perimeter/plant projected area. **(D)** The *jaz1* mutant lines showed increased drought resistance. PAR **(E)** and RWC **(F)** of the *jaz1* mutant lines and Dongjin under normal and drought stress conditions. Error bars represent the SE of three replicates (^∗∗^*P* < 0.01, ^∗^*P* < 0.05, Student’s *t*-test).

Our results described above indicated that OsJAZ1 has a negative effect on drought resistance at the seedling stage. Therefore, we wondered whether JAZ1 affects drought resistance at the reproductive stage. We conducted a drought resistance phenotyping of the *OsJAZ1*-OE lines and T-DNA mutant lines along with their WT controls (ZH11 and Dongjin) at the panicle development reproductive stage by using the high-throughput rice phenotyping facility (HRPF) (Yang et al., 2014). The dynamic changes of the plants to drought stress treatment were recorded by RGB digital cameras (**Figures 3A,D**) installed in the facility. As shown in **Figure 3B**, an image index named PAR (plant perimeter divided by projected area), which mainly reflects the degree of leaf-rolling (see description in Materials and Methods), was significantly greater in the *OsJAZ1*-OE plants than the WT ZH11 under drought stress conditions. This result indicated that the OE plants were more sensitive to drought stress treatment. The RWC is a commonly used indicator for the evaluation of the degree of cell and tissue hydration. As shown in **Figure 3C**, the RWC values of the *OsJAZ1*-OE plants (OE-1 and OE-2) were significantly lower (59.36% and 65.21%) than the WT ZH11 (82.15%) under drought stress conditions. This result further supported the negative effect of *OsJAZ1* in drought resistance. For the *jaz1* mutant lines, the PAR was significantly lower compared to the WT Dongjin under drought stress conditions, while the RWC values of the *jaz1* mutant lines (mut-1 and mut-2) were significantly greater (83.38% and 79.44%) than the WT (61.88%) under drought stress conditions (**Figures 3E,F**). At the same time, we performed drought testing in a paddy field facilitated with a movable rain-off shelter. We found that the green leaves of the *OsJAZ1*-OE plants were significantly fewer than that of ZH11 after severe drought stress treatment. However, we did not observe a significant difference in the drought sensitivity phenotype between the *jaz1* mutant and the WT in the field at the reproductive stage (Supplementary Figure 1). However, there were no differences in yield-related traits between the transgenic and WT plants under drought stress conditions, which may be mainly due to the fact that OsJAZ1/EG2 has been reported with a role in the regulation of spikelet development (Cai et al., 2014), and so the ectopic expression or mutation of this gene may lead to abnormal fertility.

### Sensitivity of *OsJAZ1*-Overexpression and Mutant Plants to ABA and MeJA Treatment

**FIGURE 4:**
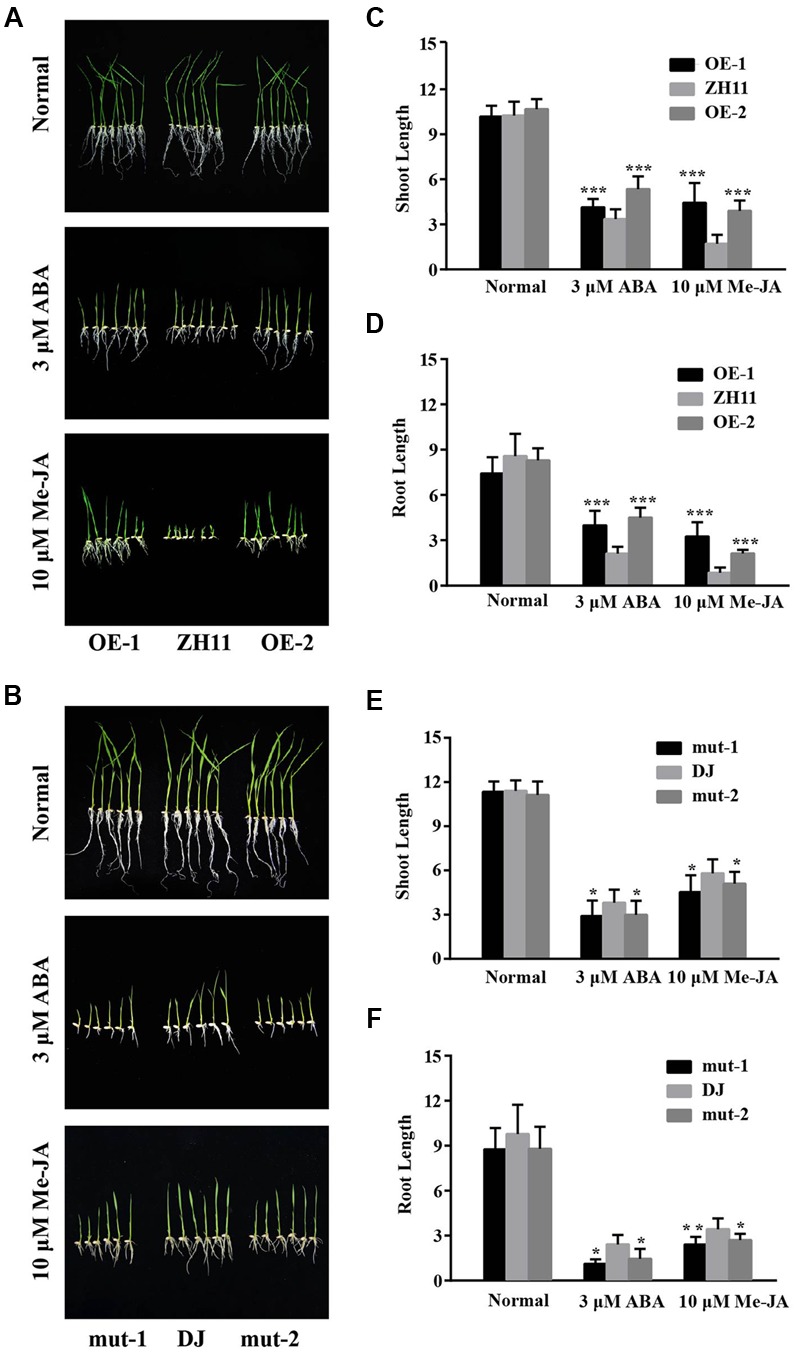
ABA and MeJA sensitivity assay of the *OsJAZ1*-OE and T-DNA mutant plants. **(A,B)** The OE lines and the mutant lines were treated with 3 μM ABA and 10 μM MeJA, under normal conditions (no addition of ABA or MeJA) as a control. **(C–F)** Shoot and root length of the seedlings were measured to estimate the sensitivity of the WT and transgenic plants. Error bars represent the SE of three replicates (^∗∗∗^*P* < 0.005, ^∗∗^*P* < 0.01, ^∗^*P* < 0.05, Student’s *t*-test).

To investigate the possible roles of OsJAZ1 in ABA and JA signaling, the *OsJAZ1*-OE plants were treated with 10 μM MeJA and 3 μM ABA. As shown in **Figure 4A**, the *OsJAZ1-*OE lines exhibited hyposensitivity to MeJA and ABA. This result was consistent with a previous study that showed *Arabidopsis* JAZ9-OE plants were insensitive to MeJA (Yang et al., 2012). Moreover, the shoot and root length of the *OsJAZ1*-OE lines were significantly longer than that of WT ZH11 under MeJA and ABA treatments (**Figures 4C,D**), but there was no significant difference under normal conditions. Therefore, we proposed that JAZ1 might repress ABA and JA signaling. To further confirm this, the *jaz1* mutant seeds were germinated for MeJA and ABA sensitivity analysis. We found that the germination of *jaz1* mutant seeds were more sensitive to MeJA and ABA compared to the WT Dongjin (**Figure 4B**). In addition, the shoot and root length of the *jaz1* mutant lines were much shorter than that of the WT Dongjin under the MeJA and ABA treatments (**Figures 4E,F**). Nevertheless, there was no significant difference in these phenotypes under normal germination conditions. Taken together, our results suggest that JAZ1 may negatively modulate ABA and JA signaling.

### Subcellular Localization of OsJAZ1

**FIGURE 5:**
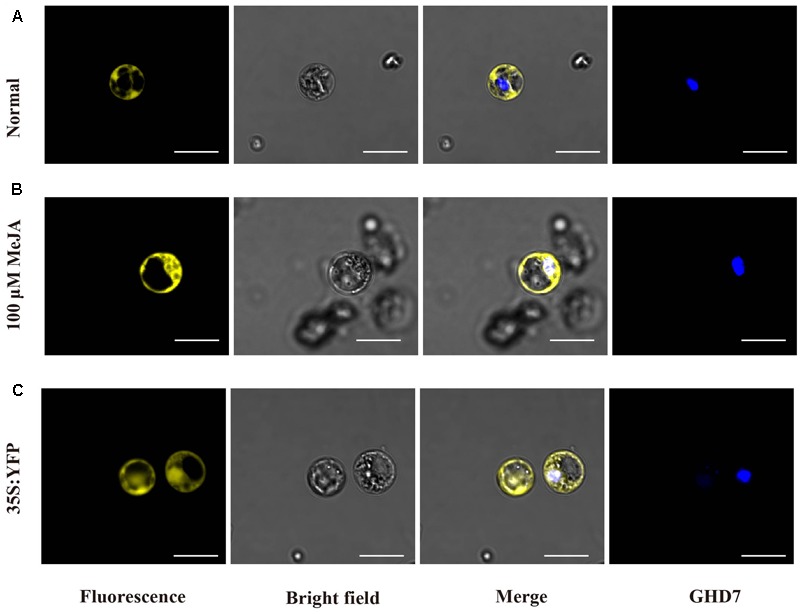
OsJAZ1 subcellular localization. **(A,B)** Confocal microscopy of OsJAZ1-YFP localization in rice protoplasts under normal conditions and 100 μM MeJA treatment, respectively. **(C)** 35S: YFP (PM999-YFP) was transformed into rice protoplasts as a control. GHD7 was used as a nucleus marker. Bars = 20 μm.

In previous studies, JAZ proteins, such as JAZ9 and JAZ10, were reported to locate in nucleus (Chung and Howe, 2009; Ye et al., 2009; Withers et al., 2012). Therefore, we wondered whether OsJAZ1 is also a nuclear protein. To investigate the subcellular localization of the OsJAZ1, the coding region of *OsJAZ1* was cloned into PM999-35 vector with C-terminal YFP tag. The tag-fused construct (35S: JAZ1-YFP) and the nuclear marker 35S: CFP-GHD7 (Xue et al., 2008) were used for transient transformation of protoplast in rice (Xie and Yang, 2013). To our surprise, OsJAZ1-YFP was found to be localized in cytoplasm (**Figure 5A**), and the YFP signal of OsJAZ1 was completely separated from the nuclear signal. In addition, YFP alone was observed in both nucleus and cytoplasm in a diffuse manner (**Figure 5C**). As we know, MYC2, a bHLH TFs, interacts with JAZ1 in nucleus (Cai et al., 2014). To further examine whether OsJAZ1 is also localized in nucleus in presence JA signaling, the protoplasts contained the construct 35S: OsJAZ1-YFP were treated with 100 μM MeJA for 3 h. As shown in **Figure 5B**, OsJAZ1-YFP was obviously observed in the nucleus and was co-localized with Ghd7 which has been reported as nuclear protein in rice. These results indicate that OsJAZ1 can function as a nuclear protein in the presence JA signaling.

### Transcriptome Analysis of the *OsJAZ1*-Overexpressed Plants

**FIGURE 6:**
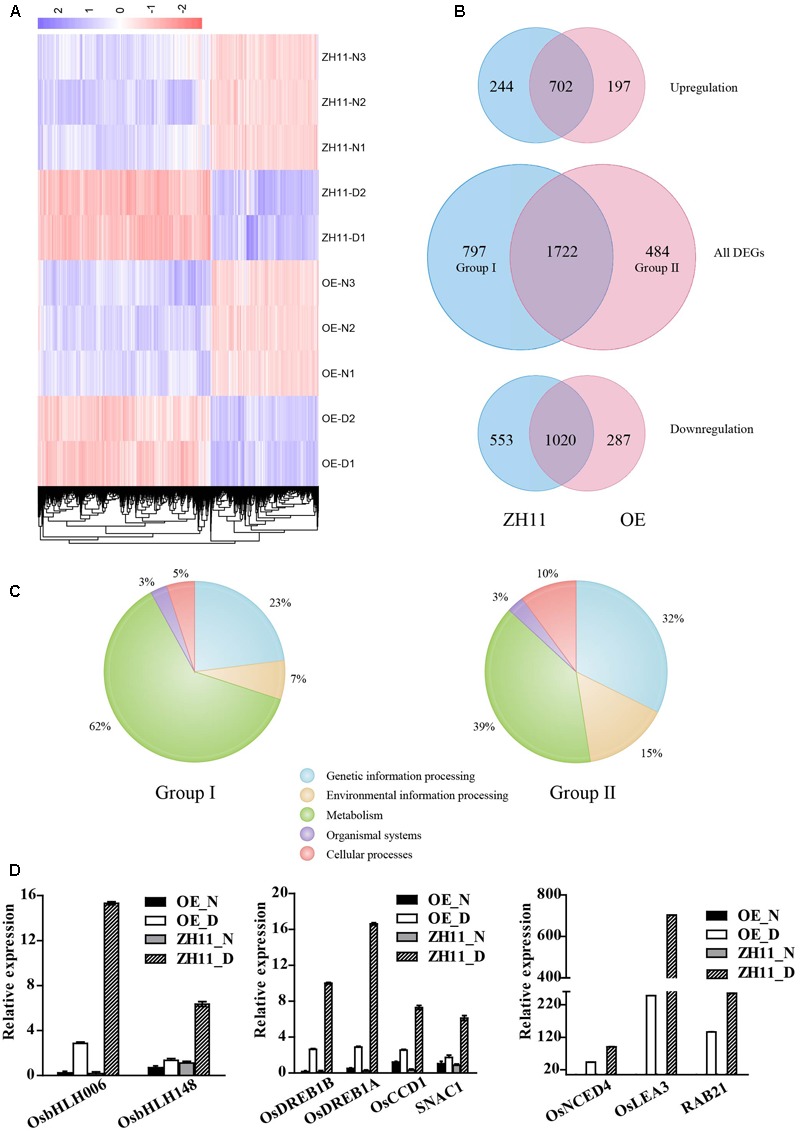
Transcriptome profiling of *OsJAZ1*-OE plants. **(A)** The expression patterns of the DEGs under normal (–N) and drought stress conditions (–D). Color scale is in log_2_ of the fold change. **(B)** Number of specific and common drought-responsive DEGs in the WT and OE plants. **(C)** KEGG classification of the specific drought-responsive DEGs (Group I and Group II) in the WT and *OsJAZ1*-OE plants. **(D)** Confirmation of several genes involved in the ABA and JA pathways and stress resistance showing lower drought-induced levels in the *OsJAZ1-*OE plants than in WT ZH11. Error bars represent the SE of three replicates.

To investigate the transcriptomic changes that may explain the drought-hypersensitive phenotype of the *OsJAZ1*-OE plants, the whole genome expression profiles of *OsJAZ1*-OE and WT ZH11 plants under normal and drought stress conditions were analyzed by RNA-seq. Each sample was collected with three biological replicates. We set the threshold as FDR < 0.05 between sample replicates, and |log_2_(fold change)|≥ 1 relative to the respective control. As a result, 2,206 and 2,519 DEGs were detected in the *OsJAZ1* OE and WT ZH11 plants, respectively, under drought stress conditions compared to normal conditions (Supplementary Files 2, 3). As shown in **Figure 6A**, the heat map revealed the different expression patterns of the DEGs (Supplementary File 1) in the *OsJAZ1*-OE and ZH11 plants. There were 899 and 946 up-regulated DEGs, and 1,307 and 1,573 down-regulated DEGs in the *OsJAZ1*-OE and WT ZH11 plants, respectively. Gene ontology (GO) enrichment analysis was conducted to identify the major functional gene groups of the DEGs. We found that most GO enrichments of the DEGs belonged to the ‘biological function’ super-group, both in the *OsJAZ1*-OE and WT ZH11 plants (Supplementary Files 2, 3). As shown in **Figure 6B**, there were 484 (Group II) and 797 (Group I) DEGs (Supplementary Files 4, 5) specifically in *OsJAZ1*-OE and WT ZH11, respectively, and the expression levels of most genes (1,722) were changed both in *OsJAZ1*-OE and WT ZH11. Among the *OsJAZ1*-OE or WT ZH11-specific DEGs, 197 and 244 DEGs were up-regulated, and 287 and 553 DEGs were down-regulated in the *OsJAZ1*-OE and WT ZH11 plants, respectively. It is noticeable that the number of up-regulated and down-regulated DEGs in the *OsJAZ1*-OE plants were much less than that in the WT ZH11, indicating that JAZ1 may repress some of the expression levels of drought-responsive genes.

To gain insights into the putative functions of the specific DEGs, GO enrichment and KEGG analyses were conducted for the DEGs in Group I and Group II. According to the GO term enrichment, the top three terms in Group I were ‘response to oxygen-containing compound’ (GO:1901700), ‘response to nitrate’ (GO:0010167), and ‘response to oxidative stress’ (GO:0006979), while the top three categories in Group II were ‘cell proliferation’ (GO:0008283), ‘regulation of DNA replication’ (GO:0006275), and ‘histone H3-K9 methylation’ (GO:0051567) (Supplementary Files 4, 5). In addition, there were 49 DEGs and 32 DEGs, respectively, in the WT ZH11 and *OsJAZ1-*OE plants belonging to the GO of ‘water deprivation’ (GO:0009414). According to the results of the KEGG analysis (Supplementary Files 4, 5), 58 and 99 DEGs were identified, respectively, in Group II and Group I. According to the KEGG analysis, 62% and 7% of the DEGs in Group I belong to ‘metabolism’ and ‘environmental information processing’ (Plant hormone signal transduction), while 39% and 15% of the DEGs in Group II belong to these two classifications, respectively (**Figure 6C**). These results suggested that the DEGs in the plant metabolism and hormone signaling pathways may mainly contribute to the hypersensitive phenotype of the *OsJAZ1-*OE plants. To further confirm this speculation, we performed quantitative real-time PCR to examine the expression profiles of some DEGs belonging to plant hormone metabolism and signaling pathways. As shown in **Figure 6D**, we found that the expressions levels of *OsNCED4* (ABA biosynthesis), *RAB21* and *OsLEA3* (typical downstream genes of ABA signaling), *OsbHLH006* and *OsbHLH148* (related to JA signaling), and *OsDREB1A, OsDREB1B, SNAC1*, and *OsCCD1* (well-known drought responsive genes) were significantly lower in the *OsJAZ1*-OE plants than in the WT ZH11 under drought stress conditions. These results indicate that the JA and ABA signaling pathways might be attenuated in the *OsJAZ1*-OE plants under drought stress conditions, and the expression levels of some drought resistance-related genes were also repressed to some extent. Together with the hyposensitive and hypersenstive phenotypes for the *OsJAZ1*-OE and the *jaz1* mutant, respectively, under MeJA and ABA treatments, these results suggest that JAZ1 may affect drought resistance partially in an ABA and JA-dependent manner.

## Discussion

Phytohormones play important roles in promoting the adaptation of plants to various abiotic stresses. The roles of JA and ABA under abiotic stress have been intensively studied. However, JAZ proteins act as a repressor of JA signaling, and there are only a few reports about the roles of JAZ proteins under abiotic stress treatment in rice (Hu et al., 2013; Wu et al., 2015; Lv et al., 2017). Here, we found that the *jaz1* mutant plants showed increased drought resistance at the seedling and reproductive stages, while the *OsJAZ1*-OE plants were more sensitive to drought stress treatment compared to the WT plants.

### *OsJAZ1* Negatively Regulates JA and ABA Signaling

According to previous studies, JAZ proteins play a negative role for JA perception and the regulation of JA responsive genes. JA-insensitive and hypersensitive mutants contributed to confirming the negative role of JAZ proteins in JA signaling. MeJA treatment was performed to examine the JA responsiveness of *OsJAZ1* (**Figure 1A**). The changed expression level of *OsJAZ1* showed that *OsJAZ1* was responsive to JA. A MeJA sensitivity test for the *OsJAZ1*-OE lines and *jaz1* mutant lines suggests that the *OsJAZ1*-OE plants were hyposensitive to MeJA (**Figure 4A**), whereas the *jaz1* mutant plants were hypersensitive to MeJA (**Figure 4B**). The result is consistent with previous results that the JAZ overexpression plants showed a hyposensitive phenotype under MeJA treatment (Chini et al., 2007; Thines et al., 2007). The expression level of *OsJAZ1* was much greater in the overexpression plants than that in WT ZH11, resulting in stronger repression on the activation of JA-responsive TFs. Therefore, we propose that *OsJAZ1* acts as a repressor in the JA signaling pathway.

Previous studies have reported that the ABA-dependent signaling pathway and the JA dependent pathway have common TFs (MYB/MYC), such as OsMYC2 (Abe et al., 2003; Adie et al., 2007). However, crosstalk between the JA and ABA signaling pathways is still ambiguous, since both synergistic and antagonistic interactions have been reported. In our study, we found that *OsJAZ1* is also significantly induced by ABA (**Figure 1C**), and a hyposensitive phenotype is present in the *OsJAZ1*-OE plants under ABA treatment (**Figure 4A**). What’s more, the *jaz1* mutant lines were hypersensitive to ABA (**Figure 4B**), indicating that *OsJAZ1* plays a negative role in the ABA signaling pathway. Our finding that *OsJAZ1* negatively regulates both the JA and ABA signaling pathways may contribute to the study of crosstalk between JA and ABA signaling. Nevertheless, the phenotypic changes of the *jaz1* mutant lines under the MeJA and ABA treatments were less obvious than that of the *OsJAZ1*-OE plants, which may be due to potential functional redundancy of OsJAZ1 homologs in rice.

### *OsJAZ1* Negatively Modulates Drought Resistance

Plant adaptation to variable environmental stresses relies on arrays of signaling networks. JAs play important roles in plant adaptation and mediate several biotic responses, plant growth and developmental processes, and recently they have been reported to regulate tolerance to abiotic stresses. The JAZ proteins, belonging to the TIFY plant-specific family, are key components of the JA signaling pathway. A previous study indicated that JAZ9 repressed the expression of *OsbHLH062* and *OsMYB30*, resulting in increased salt and cold tolerance (Wu et al., 2015; Lv et al., 2017). However, there is no direct evidence that JAZ proteins can affect drought resistance. In this study, we found that the *jaz1* mutant lines showed increased drought resistance compared to the Dongjin control rice at both the seedling and reproductive stages (**Figures 2D, 3D**). As expected, the *OsJAZ1*-OE plants exhibited decreased drought resistance at both the seedling and reproductive stages (**Figures 2E, 3A**). In addition, we also tested the drought resistance of the *OsJAZ1* transgenic rice in the field. The results showed that the *OsJAZ*1-OE lines were more sensitive to drought stress treatment in terms of the green leaves which remained after drought recovery, which is consistent with the increased drought sensitivity of the *OsJAZ*1-OE lines observed at the seedling stage. However, the *jaz1* mutant lines in the field did not show an obvious hyposensitive phenotype as we expected (Supplementary Figure 1). One of the possible reasons may be that the suppression of *OsJAZ*1 only has a relatively small genetic effect in changing drought resistance, especially under the severe drought stress treatment we applied in this experiment. Nevertheless, it cannot be excluded that other homologs of OsJAZ1 may have similar functions in regulating drought resistance. In addition, we suspect that the overexpression or mutation of *OsJAZ1* may cause fertility abnormalities, since this gene has been reported with a role in the control of spikelet development (Cai et al., 2014). Therefore, the relative yield or spikelet fertility are not suitable traits to evaluate the effect of this gene on drought resistance at the reproductive stage.

We conducted transcriptome profiling to find clues to the mechanisms responsible for the drought-hypersensitive phenotype of the *OsJAZ1*-OE plants. According to the profiling results, we speculate that the hypersensitive phenotype of the *OsJAZ1*-OE plants under drought stress treatment may be partially attributed to the significantly decreased expression levels of abiotic stress and ABA and JA signaling and stress-responsive genes. These include *OsNCED4, OsLEA3, RAB21, OsbHLH006, OsbHLH148, OsDREB1A, OsDREB1B, SNAC1*, and *OsCCD1* which were confirmed by qRT-PCR analysis. *OsNCED4, OsLEA3*, and *RAB21* are well known ABA and abiotic stress responsive genes (Ganguly et al., 2012). Furthermore, *OsNCED4* is a key gene in ABA biosynthesis and involved in the regulation of drought resistance (Zong et al., 2016). *OsLEA3* encodes a group 3 late-embryogenesis abundant protein and plays an important role in drought and salt stress responses (Hu, 2008). The suppressed expression of *OsNCED4, OsLEA3*, and *RAB21* may result in an attenuation of the ABA pathway and subsequently lead to the drought-hypersensitive phenotype of the *OsJAZ1*-OE plants. Furthermore, no DEGs in Group II were enriched in the GO terms related to ABA signaling (Supplementary File 4), such as ‘response to ABA’ (GO:0009737) and ‘regulation of ABA biosynthetic process’ (GO:0010115). These results further suggest that ABA signaling was suppressed to some extent in the process of OsJAZ1-regulated drought resistance.

Previous studies have shown that JA also plays an important role in abiotic stress resistance. In this study, the expression levels of *OsbHLH006* and *OsbHLH148* were repressed in the *OsJAZ1*-OE plants under drought stress treatment. OsbHLH006 and OsbHLH148 are both basic helix-loop-helix proteins that regulate drought resistance via the JA-dependent pathway (Seo et al., 2011; Miyamoto et al., 2013). Previous studies have reported that *OsDREB1A, OsDREB1B, SNAC1*, and *OsCCD1* positively regulate drought resistance in rice (Dubouzet et al., 2003; Figueiredo et al., 2012; Redillas et al., 2012; Jing et al., 2016). *OsDREB1A* and *OsDREB1B* regulate the expression of abiotic stress-related genes via an ABA-independent pathway. Here, we showed that the expression levels of *OsDREB1A, OsDREB1B, SNAC1*, and *OsCCD1* were suppressed in the *OsJAZ1*-OE plants under drought stress conditions, which further supported the hypersensitive phenotype in the *OsJAZ1*-OE plants under drought stress treatment. Besides these reported genes related to the JA and ABA pathways or drought resistance, there are many DEGs in Group I enriched in several GO terms related to other stress responses, such as ‘response to oxidative stress’ (GO:0006979), ‘response to water deprivation’ (GO:0009414), and ‘response to salt stress’ (GO:0009651), while there were none or fewer DEGs in Group II enriched in these GO terms.

## Conclusion

We have demonstrated that OsJAZ1 functions as a negative regulator in the drought resistance of rice, partially in an ABA-dependent and JA-dependent manner.

## Author Contributions

JF designed and performed the experiments and wrote the manuscript. HW generated the transgenic materials and performed the experiments. SM, DX, and RL provided assistance with subcellular localization and data analysis. LX designed the experiments and wrote the manuscript.

## Conflict of Interest Statement

The authors declare that the research was conducted in the absence of any commercial or financial relationships that could be construed as a potential conflict of interest.

## Footnotes

1 http://www.postech.ac.kr/life/pfg/risd/

2 http://www.biocloud.net/

3 http://www.postech.ac.kr/life/pfg/risd/
