# Supplementary material for: OsJAZ1 Attenuates Drought Resistance by Regulating JA and ABA Signaling in Rice
Source: Front Plant Sci. 2017 Dec 11;8:2108. doi: 10.3389/fpls.2017.02108 (PMC5733117; doi:10.3389/fpls.2017.02108)
Supplement: Supplementary file 1 [file Table_1.PDF]

Supplementary Table 1. Primers used in this study

Primers Name	Primers sequence (5’ to 3’)	Purpose
OsbHLH006F	GCGCGAACAAGAACATCCTC	qRT-PCR
OsbHLH006R	TTGATGATCGACGCCTTGTCC	qRT-PCR
OsbHLH148F	AACGTCGAGACCACGATTGC	qRT-PCR
OsbHLH148R	TGTCAGGCTGGTTCCTCTCTACT	qRT-PCR
OsNCED4F	GATTGCACGGCACCTTCATT	qRT-PCR
OsNCED4R	CTCTGTAATTTGATTTTTCACTGGCTAAT	qRT-PCR
OsLEA3F	GGTGATGTGTACTGATGATGTT	qRT-PCR
OsLEA3R	ACAAATGCGGGCTTTAGG	qRT-PCR
RAB21F	CACACCACAGCAAGAGCTAAGTG	qRT-PCR
RAB21R	TGGTGCTCCATCCTGCTTAAG	qRT-PCR
OsDREB1BF	CTCGACGACGGGTTCAGGTTC	qRT-PCR
OsDREB1BR	CGTCCTCCCACCACGCTC	qRT-PCR
OsDREB1AF	CGAGGAGTCCGCCGCCACC	qRT-PCR
OsDREB1AR	TCCATGAGCATCCCCTGCGCCAA	qRT-PCR
OsCCD1F	GGACTACCTGCCGGTGAT	qRT-PCR
OsCCD1R	CTGTCGAAGGTGATGAGCCC	qRT-PCR
OsNAC9F	GGATCTCTCAGGTTGGATGATTG	qRT-PCR
OsNAC9R	TTCCCCTGCTGCATCTTCTC	qRT-PCR
JAZ1rtF	CAGCAGGTTGGTGTACAAATGC	qRT-PCR
JAZ1rtR	TCCATCCCTGATGCTTCCAT	qRT-PCR
Z1-mutF	GAGTCACGGTTGAAATCTTG	PCR
Z1-mutR	GCAACTAGAATGACACCCTAA	PCR
PGAR	TTGGGGTTTCTACAGGACGTAAC	PCR
