# Supplementary material for: OsJAZ1 Attenuates Drought Resistance by Regulating JA and ABA Signaling in Rice
Source: Front Plant Sci. 2017 Dec 11;8:2108. doi: 10.3389/fpls.2017.02108 (PMC5733117; doi:10.3389/fpls.2017.02108)
Supplement: Supplementary file 2 [file Presentation_1.PDF]

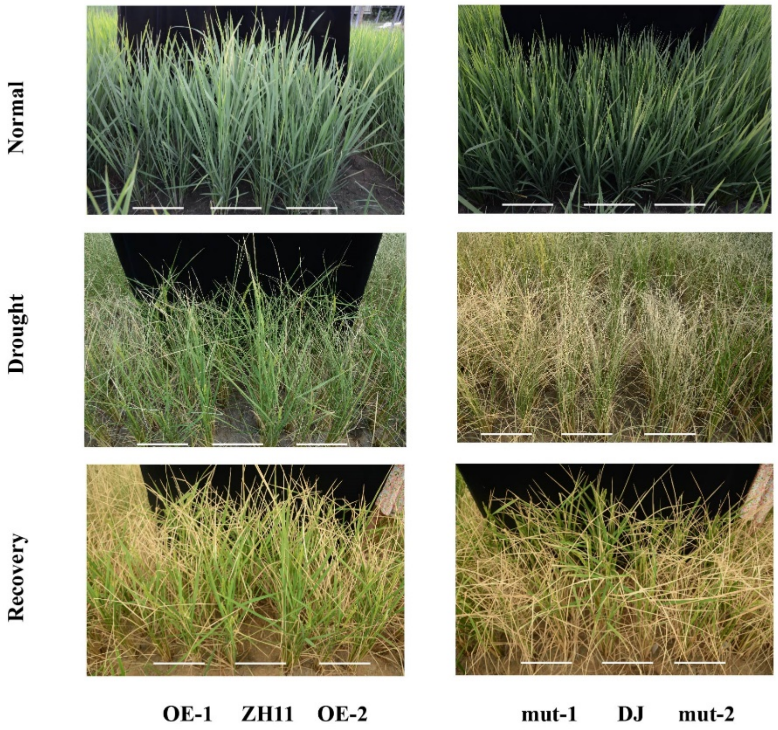

Supplementary Figure 1. Drought resistance analysis of the transgenic and wild-type plants in a refine paddy field facilitated with a movable rain-off shelter.
